# Outcomes and standardized tools in telehealth physical therapy for children with cerebral palsy: A scoping review using the ICF framework

**DOI:** 10.1111/dmcn.70006

**Published:** 2025-10-17

**Authors:** Isabella S. Christovão, Paula S. de C. Chagas, Ana Alice V. Aniceto, Daiane A. de O. Bettoni, Lorena C. Ferreira, Hércules R. Leite, Ana Cristina R. Camargos

**Affiliations:** ^1^ Graduate Program in Rehabilitation Sciences, School of Physical Education, Physical Therapy and Occupational Therapy, Universidade Federal de Minas Gerais Belo Horizonte Brazil; ^2^ Graduate Program in Rehabilitation Sciences and Physical and Functional Performance, Faculty of Physical Therapy, Universidade Federal de Juiz de Fora Juiz de Fora Brazil; ^3^ Department of Physical Therapy School of Physical Education, Physical Therapy and Occupational Therapy, Universidade Federal de Minas Gerais Belo Horizonte Brazil

## Abstract

**Aim:**

To identify the outcomes and standardized tools used to measure changes following telehealth‐delivered physical therapy interventions in children and young people with cerebral palsy, within the framework of the International Classification of Functioning, Disability and Health domains, and to describe how these tools were administered.

**Method:**

This scoping review followed the steps of the JBI and identified studies on telehealth‐delivered physical therapy interventions in children and young people up to 20 years old using standardized assessment tools. Searches were conducted in MEDLINE/PubMed, Embase, Cochrane, Scopus, Web of Science, PEDro, Lilacs, and grey literature, with no date or language restrictions.

**Results:**

Fourteen studies (625 participants, age range 6 months–20 years, all levels of the Gross Motor Function Classification System) met inclusion criteria. Outcomes were primarily in the activity domain (59.3%), followed by body structure and function (29.6%) and participation (22.2%) domains. Thirty standardized tools were used; the most frequent were the Assisting Hand Assessment (*n* = 5) and Gross Motor Function Measure (*n* = 3). Most standardized tools were applied in‐person (68.2%), while others used mixed methods (telehealth and in‐person: 18.2%) or did not specify the mode of administration (6.8%). Only two studies administered all tools by telehealth.

**Interpretation:**

The outcomes and standardized tools identified in this review target the activity domain, reflecting parental priorities. In‐person assessments remain the preferred modality for conducting standardized evaluations. Further research is necessary to investigate the feasibility and measurement properties of using standardized tools by telehealth.

## Graphical abstract

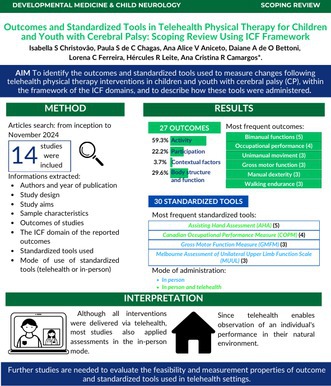

Plain language summary: https://onlinelibrary.wiley.com/doi/10.1111/dmcn.70070

**Abbreviations**
GMFM: Gross Motor Function Measure
ICF: International Classification of Functioning, Disability and Health.
10MWT: 10‐metre walk test
1MWT: 1‐minute walk test
6MWT: 6‐minute walk test

**What this paper adds**

- Standardized tools were mostly used to assess activity.
- Most standardized tools were administered in‐person.
- Standardized tools for telehealth lack comprehensive evaluation of measurement properties.

In recent years, telehealth has emerged as a promising approach for delivering rehabilitation interventions, with its use experiencing a substantial rise since the onset of the COVID‐19 pandemic.^1^ As the COVID‐19 pandemic advanced and social distancing became essential, healthcare professionals sought safe and effective modes to sustain their services.^2^ Before the pandemic, approximately 2% of physical therapists reported using telehealth services; however, 1 year after the pandemic's onset, this percentage had increased to 48%.^3^

Healthcare by telehealth involves assessment and intervention and is defined as the provision of rehabilitation services in a remote location with the use of the telecommunication technologies, such as video conferencing, online platforms, and mobile applications,^4^ for assessing and monitoring individuals' physical impairments, activity limitations, and participation restrictions.^5^ Cerebral palsy (CP), a permanent neurodevelopmental disorder characterized by activity limitations,^6^ can be addressed through telehealth‐delivered physical therapy interventions implemented by parents in a home setting on the basis of the principles of active motor learning.^7^, ^8^, ^9^ Delivering interventions by telehealth might decrease healthcare costs by reducing the need for travel to in‐person appointments.^1^ Telehealth enhances access to services for families residing in remote areas or regions with limited healthcare availability^10^ and may enable continuity of care during public health emergencies.^11^ However, several barriers to implementation exist, including limited access to reliable Internet and technology, as well as the challenges families encounter in understanding and performing home‐based activities.^12^

An important challenge has been the selection of appropriate tools to accurately assess clinically relevant outcomes in this modality.^11^, ^13^ Standardized tools are defined as structured assessment instruments developed through scientifically rigorous methods to consistently and objectively measure specific constructs and/or outcomes^14^ and assist professionals in identifying clinically significant changes for children and their families.^15^, ^16^ Several standardized tools are recommended for evaluating the outcomes of an intervention.^16^ However, therapists must ensure that the chosen tool and its components accurately represent the construct or outcome of interest. This requires evaluating whether the tool aligns with outcomes that are important to patients and caregivers, ensuring its practicality and ease of use in the intended context, and verifying its suitability for the target population.^15^

A recent systematic review evaluated the validity, reliability, and clinical utility of telehealth physiotherapy assessments for children and adults with and without motor impairments; however, no studies involving children with CP were included.^11^ Identifying outcomes and standardized tools used to assess clinical changes in children and young people with CP following telehealth‐delivered physical therapy interventions can enhance the efficiency, relevance, and overall quality of clinical practice and research focused on this population.^17^ Moreover, mapping these tools using the International Classification of Functioning, Disability and Health (ICF) framework lens^18^ can offer a comprehensive understanding of the extent to which various domains of functioning are addressed.

Thus, this scoping review aims to identify the main outcomes and standardized tools used to measure changes following telehealth‐delivered physical therapy interventions in children and young people with CP, with reference to the domains of the ICF framework. The specific objectives were to (1) identify outcomes and standardized tools used to measure changes after telehealth‐delivered physical therapy interventions; (2) analyse the content of outcomes within the framework of the ICF domains; and (3) identify the mode of application of the standardized tools (in‐person or by telehealth).

## METHOD

This scoping review adhered to the methodological framework of the JBI^19^ and was prospectively registered in the Open Science Framework Register (https://doi.org/10.17605/OSF.IO/JXCVH). The review followed the statement of Preferred Reporting Items for Prism Extension for Scoping Reviews (PRISMA‐ScR).^20^

### Search strategy

The search strategies were done in Medline/PubMed, Embase, Cochrane, Scopus, Web of Science, PEDro, Lilacs, and grey literature, and researched on Google and Google Scholar. The descriptors used in our research were (‘Cerebral Palsy’) AND (‘Physical Therapy Specialty’ OR ‘Physical Therapists’ OR ‘Rehabilitation’ OR ‘Exercise Therapy’ OR ‘Neurological Rehabilitation’ OR ‘Rehabilitation Exercise’) AND (‘Telemedicine’ OR ‘Telehealth’ OR ‘eHealth’ OR ‘mHealth’). A detailed list of the descriptors and search strategy for each database is provided in Table S1. We also manually searched the identified literature for possible additional studies. The search strategy (without date restrictions) was conducted in November 2024 and researchers were assisted by a librarian.

### Inclusion and exclusion criteria

This scoping review included randomized controlled trials, non‐randomized controlled trials, single‐subject designs, case studies, and case series/case reports that investigated telehealth‐delivered physical therapy interventions and the use of standardized tools for assessment in children and young people with CP up to 20 years old. Only full‐text studies, without language restrictions, were included in the study.

Services specific to other specialties were excluded unless they were combined with physical therapy (e.g. occupational therapy). Additionally, studies that did not provide a definition of the tools used in their methods were also excluded. Conference abstracts, research letters, editorials, opinions, letters to editors, notes or project evaluation reports, protocol studies, epidemiological studies, and synthesis of knowledge (e.g. narrative, systematic, and scoping reviews) were excluded.

### Screening

The screening of identified titles and abstracts, and potential full texts, was conducted independently by two reviewers (ISC and LCF). The eligibility criteria were piloted on the first five records to ensure clarity and consistency. Disagreements at any stage were resolved with a third reviewer (ACRC), when necessary.

### Data extraction

The data extraction included authors and year of publication, study design, study aims, sample characteristics, outcomes of studies, the ICF domain of the reported outcomes, standardized tools used, and mode of use of standardized tools (telehealth or in‐person).

The content of the outcomes was classified according to the domains defined by the ICF framework. The ICF provides a comprehensive framework for understanding the multidimensional aspects of health and disability. These domains include (1) body structures and functions, which focuses on the physiological and anatomical aspects of the body, such as sensory functions, muscle strength, and joint mobility; (2) activities, which considers the individual's ability to perform tasks in activities, such as self‐care, mobility and communication; (3) participation, which considers involvement in a life situation; (4) environmental factors, which includes external influences that can either facilitate or hinder an individual's functioning; and (5) personal factors, which includes characteristics such as age, sex, coping styles, beliefs, and education.^18^ When appropriate, outcomes were classified under more than one domain. The ICF domains were extracted by two independent reviewers (ISC and LCF). Interrater reliability was assessed using the weighted kappa statistic, yielding a high level of agreement (*κ* = 0.90; *p* < 0.001). Subsequently, three additional researchers (PSCC, HRL, and ACRC) independently reviewed and verified the domain classifications to ensure consistency and accuracy.

## RESULTS

The database searches identified 1207 studies; after removing duplicates and screening the title and abstract, 95 full‐text studies were evaluated under our eligibility criteria. The flow of identification and screening of studies, including reasons for exclusion, are illustrated in Figure S1. As shown, 14 studies were included in this scoping review.

The studies were published between 2015 and 2024. Seven studies (50%) were conducted before the COVID‐19 pandemic and seven studies (50%) during or after the declaration of it. Twelve studies (85.7%) implemented exclusively telehealth‐delivered physical therapy interventions, while two (14.3%) used hybrid interventions, incorporating both in‐person and telehealth components for the same group or participant.

The study design is described in Table 1. A total of nine randomized controlled trials were identified, including one randomized controlled trial protocol and one parallel‐arm pilot study. The review also incorporated two non‐randomized studies and three case series studies.

**TABLE 1:** Main characteristics of the studies included in this scoping review.

Study	Study design	Study aims	Sample characteristics
James et al.^23^	Randomized controlled trial	Investigate the effectiveness of a Web‐based therapy programme, ‘Move it to improve it’ (Miti), in children with unilateral CP on occupational performance, upper limb function, and visual perception.	Children (*n* = 101) with spastic type unilateral CP, MACS levels I–III and GMFCS levels I or II, aged 8–18 years with sufficient cooperation and cognitive understanding to perform required tasks, and Internet access at home.
Mitchell et al.^28^	Randomized controlled trial	Determine the efficacy of Web‐based training on activity capacity and performance in children with unilateral CP.	Children and adolescents (*n* = 101) aged 8–17 years with unilateral CP, GMFCS levels I and II and MACS levels I–III.
Ferre et al.^22^	Randomized controlled trial	Examine the efficacy of caregiver‐directed, home‐based intensive bimanual training in children with unilateral spastic cerebral CP.	Experimental group: 12 children, aged 2 years 6 months to 10 years 1 month Control group: 12 children, aged 2 years 6 months to 10 years 1 month
Surana et al.^9^	Randomized controlled trial	Determine the effectiveness of lower‐extremity functional training compared with an attention control group receiving upper‐extremity bimanual training (hand–arm bimanual intensive therapy).	Experimental group: 12 children; 5.8 (±2.3) years Control group: 12 children; 5.1 (±2.6) years Both groups GMFCS levels I and II, ability to walk independently, ability to follow two‐step instructions and complete testing, and ability of caregiver to provide one‐to‐one attention to the child during daily activities.
Pietruszewski et al.^29^	Randomized controlled trial (protocol)	Document treatment fidelity and provide initial testing of telehealth intervention delivery in a new subject sample.	Experimental group: 7 children; control group: 6 children; 6–24 months old Diagnosis of hemiplegic or asymmetric quadriplegic CP; asymmetric CP using Hammersmith Infant Neurological Examination Asymmetry Score >6; access to Internet connectivity.
Cristinziano et al.^26^	Non‐randomized	Evaluate the effects of telerehabilitation on gross motor function in children with CP during COVID‐19 lockdown.	Fifty‐three children with CP aged between 6 months and 12 years classified according to the GMFCS.
Lai et al.^30^	Randomized controlled trial (parallel‐arm pilot)	Examine the preliminary efficacy of a youth‐based adapted movement‐to‐music intervention for increasing both activity and leisure‐time physical activity participation among adolescents with CP, compared with a 4‐week waiting list control group.	Adolescents (*n* = 49); 10–19 years, with CP who walked or used wheelchairs.
Molinaro et al.^8^	Case series	Assess whether action observation treatment has the potential to improve the functional recovery of children with CP even when used at patients' homes, in a telerehabilitation setting. The focus was on the recovery of upper limb motor functions.	Children (*n* = 10), aged 5–12 years (primary school and the first level of secondary school according to the Italian school system) with a confirmed diagnosis of CP. Inclusion criteria were MACS ≥ level IV, full scale IQ > 70, absence of major visual and/or auditory deficits, well controlled seizures.
Beani et al.^21^	Randomized controlled trial	Investigate the feasibility of a new rehabilitative home‐based approach, called Tele‐UPCAT (Tele‐monitored UPper limb Children Action observation Training), based on the principles of action observation training, in a group of Italian children and adolescents with unilateral CP.	Children and adolescents (*n* = 29), age between 5 years and 20 years, confirmed diagnosis of spastic motor type of unilateral CP, minimum ability of manual function defined as the ability to passively hold an object placed in the hand or hold and stabilize an object with a hand while the other manipulates it (i.e. House Functional Classification System ≥2), a normal cognitive level (i.e. IQ ≥ 70), and no disabling behavioural disorders.
Çelikel et al.^7^	Randomized controlled trial	Assess whether the effect of a motor‐learning‐based treatment provided by a telerehabilitation method on the quality of life of children with CP during the COVID‐19 period was equivalent to face‐to‐face treatment.	Children with CP and spastic paraparesis (*n* = 25), aged 3–17 years; GMFCS levels I–II.
Reidy et al.^24^	Case series	Describe the feasibility and clinical experiences of using hybrid telehealth to deliver paediatric CIMT during the COVID‐19 pandemic. In addition, to present the outcomes of hybrid telehealth CIMT and subsequent in‐person CIMT models in two paediatric clients.	Patient 1: female infant with hemiplegic CP, 8 months. Patient 2: male infant with left hemiplegic CP and polymicrogyria, 8 months.
Rodriguez‐Costa et al.^27^	Case series	Assess whether a telecare intervention consisting of action observation training with a family‐centred approach produces improvements in functionality in children and adolescents with CP.	Seven females with CP aged between 6 years and 17 years with CP; GMFCS, MACS, and Communication Function Classification System I–III
Sel et al.^25^	Randomized controlled trial	Determine the effectiveness of usual care plus a telerehabilitation‐based structured home programme on preschool children with CP compared with usual care.	Experimental group: 24 children, aged 3–6 years (mean 4.66 ± 1.08 years) Control group: 24 children, aged 3–6 years (mean 4.66 ± 1.08 years)
Oliveira et al.^31^	Non‐randomized	Analyse the effects of an individualized telehealth home programme on the performance of functional goals of children and adolescents with CP during the COVID‐19 pandemic.	Children/adolescents with CP (*n* = 144; median age = 92 months), representing all GMFCS levels.

Abbreviations: CIMT, constraint‐induced movement therapy; CP, cerebral palsy; GMFCS, Gross Motor Function Classification System; MACS, Manual Ability Classification System.

### Characteristics of participants

Table 1 describes the main characteristics of the studies included in this scoping review. The 14 included studies involved a total of 625 children and young people with CP, aged between 6 months and 20 years, classified according to the Gross Motor Function Classification System (GMFCS) as follows: level I (23%), level II (33%), level III (5.8%), level IV (12.5%), and level V (16.8%). Two studies did not report the GMFCS level (5%), while another reported that the children were classified in GMFCS levels I and II without distinguishing between them (4%). Regarding the subtypes of CP, the studies analysed children with spastic unilateral CP (46.2%) and spastic bilateral subtype (4.3%); 49.5% of studies did not specify the subtype of CP.

### Outcomes

Twenty‐seven outcomes were reported. Sixteen (59.3%) outcomes were related to the activity, eight (29.6%) were related to body structure and function, six (22.2%) were related to participation, and one (3.7%) was related to the contextual factors domain, according to the ICF framework. The most frequently reported outcomes in the activity domain were bimanual function (35.7%),^8^, ^21^, ^22^, ^23^, ^24^ gross motor function (21.4%),^25^, ^26^, ^27^ walking endurance (21.4%),^9^, ^27^, ^28^ and manual dexterity (21.4%).^21^, ^22^, ^23^ The most frequently reported outcome in the body structure and function domain was balance (14.3%).^9^, ^27^ Detailed results are provided in Table 2.

**TABLE 2:** Outcomes, nature of the outcome according ICF framework, standardized tools, and mode of use of the standardized tools.

Study	Outcomes	Nature of outcomes according to ICF	Standardized tools	Mode of use
James et al.^23^	Occupational performance	Activity, participation	COPM	In‐person
	Daily activities performance	Activity	Assessment of Motor and Process Skills	In‐person
	Bimanual function	Activity	Assisting Hand Assessment	In‐person
	Manual dexterity	Activity	Jebsen–Taylor Test of Hand Function	In‐person
	Unimanual movement	Body structure and function, activity	MUUL	In‐person
	Visual perception	Body structure and function	Test of Visual Perceptual Skills	In‐person
Mitchell et al.^28^	Recreational participation	Participation	Assessment of Life Habits	In‐person
	Activity capacity to functional tasks	Activity	Verschuren's protocol (maximal repetitions of sit to stand, lateral step up using a 20‐cm step, and half‐kneel to standing for the dominant and non‐dominant legs over a 30‐second period)	In‐person
	Walking endurance	Activity	6MWT	In‐person
	Daily activities performance	Activity	Accelerometer	In‐person
	Mobility	Activity	28‐Item Mobility Questionnaire	In‐person
Ferre et al.^22^	Occupational performance	Activity, participation	COPM	In‐person (pre‐ intervention) and telehealth (pre‐, post‐ intervention and follow‐up)
	Bimanual function	Activity	Assisting Hand Assessment	In‐person (pre‐ intervention) and telehealth (pre‐, post‐ intervention and follow‐up)
	Manual dexterity	Activity	Box and Blocks Test	In‐person (pre‐ intervention) and telehealth (pre‐, post‐ intervention and follow‐up)
Surana et al.^9^	Walking endurance	Activity	1MWT	In‐person (pre‐ intervention) and telehealth (pre‐ and post‐ intervention)
	Walking speed	Activity	10MWT	In‐person (pre‐ intervention) and telehealth (pre‐ and post‐ intervention)
	Locomotion performance	Activity	ABILOCO‐Kids	In‐person (pre‐ intervention) and telehealth (pre‐ and post‐ intervention)
	Muscle strength	Body structure and function	30‐second chair rise test	In‐person (pre‐intervention) and telehealth (pre‐ and post‐intervention)
	Balance	Body structure and function	Single leg stance	In‐person (pre‐intervention) and telehealth (pre‐ and post‐intervention)
Pietruszewski et al.^29^	Reach smoothness	Body structure and function	Kinematic assessment (Vicon Motion Systems)	In‐person
	Motor development	Activity	Bayley‐III	In‐person
Cristinziano et al.^26^	Gross motor function	Activity	GMFM‐66	In‐person
Lai et al.^30^	Leisure‐time physical activity	Participation	CAPE	Telehealth
	Pain and fatigue	Body structure and function	Neuro‐QoL	Telehealth
Molinaro et al.^8^	Bimanual function	Activity	Assisting Hand Assessment	Not reported
	Unimanual movement	Body structure and function, activity	MUUL	Not reported
Beani et al.^21^	Participation and environmental factors	Participation, environmental factors	Participation and Environment Measure ‐ Children and Youth	In‐person
	Quality of life	Multidimensional	Cerebral Palsy Quality of Life Questionnaire	In‐person
	Manual performance	Activity	ABILHAND‐Kids	In‐person
	Bimanual function	Activity	Assisting Hand Assessment	In‐person
	Manual dexterity	Activity	Box and Blocks Test	In‐person
	Unimanual movement	Body structure and function, activity	MUUL	In‐person
Çelikel et al.^7^	Quality of life	Multidimensional	Pediatric Quality of Life Inventory	Not reported
Reidy et al.^24^	Bimanual function	Activity	Assisting Hand Assessment	In‐person
	Unimanual function	Body structure and function, activity	Quality of Upper Extremity Skills Test	In‐person
Rodriguez‐Costa et al.^27^	Gross motor function	Activity	GMFM‐88	In‐person
	Walking endurance	Activity	6MWT	In‐person
	Walking speed	Activity	10MWT	In‐person
	Balance	Body structure and function	Pediatric Balance Scale	In‐person
Sel et al.^25^	Occupational performance	Activity, participation	COPM	In‐person
	Goal achievement	Activity, participation	Goal Attainment Scaling	In‐person
	Daily functioning	Activity, participation	Pediatric Evaluation of Disability Inventory	In‐person
	Gross motor function	Activity	GMFM‐66	In‐person
Oliveira et al.^31^	Occupational performance	Activity, participation	COPM	Telehealth

Abbreviations: 10MWT, 10‐metre walk test; 1MWT, 1‐minute walk test; 6MWT, 6‐minute walk test; Bayley‐III, Bayley Scales of Infant and Toddler Development, Third Edition; CAPE, Children's Assessment of Participation and Enjoyment; COPM, Canadian Occupational Performance Measure; GMFM, Gross Motor Function Measure; ICF, International Classification of Functioning, Disability and Health; MUUL, Melbourne Assessment of Unilateral Upper Limb Function Scale.

### Standardized tools

A total of 30 different standardized tools were identified to assess outcomes across the studies. The most frequently used tool for evaluating the activity domain was the Assisting Hand Assessment,^8^, ^21^, ^22^, ^23^, ^24^ which aims to assess bimanual function, followed by the Gross Motor Function Measure (GMFM),^25^, ^26^, ^27^ used to assess gross motor function. The Assisting Hand Assessment was administered both in‐person^21^, ^22^, ^23^, ^24^ and by telehealth,^22^ whereas the GMFM was exclusively administered in‐person.^25^, ^26^, ^27^ Other standardized tools for assessing the activity domain were administered in‐person only, including the 6‐minute walk test (6MWT),^27^, ^28^ ABILHAND‐Kids,^21^ Bayley Scales of Infant and Toddler Development, Third Edition,^29^ Assessment of Motor and Process Skills,^23^ Jebsen–Taylor Test of Hand Function,^23^ Verschuren's protocol^28^, accelerometer,^28^ and the 28‐item Mobility Questionnaire.^28^ The Box and Blocks Test was administered both in‐person^21^, ^22^ and by telehealth.^22^ Additionally, the 1‐minute walk test (1MWT), 10‐metre walk test (10MWT), and ABILOCO‐Kids were administered through both in‐person and telehealth in the same study.^9^

In the body structure and function domain, balance was assessed by single leg stance^9^ and the Pediatric Balance Scale.^27^ The single leg stance^9^ was administered both in‐person and by telehealth, whereas the Pediatric Balance Scale^27^ was administered in‐person only. The Neurology Quality of Life Measurement System^30^ was administered exclusively by telehealth. The 30‐second chair rise test^9^ was also conducted both in‐person and by telehealth. Other standardized tools used to assess the body structure and function domain were administered exclusively in‐person, including kinematic measures^29^ and the Test of Visual Perceptual Skills.^23^ Two standardized tools were used to assess the participation domain: the Children's Assessment of Participation and Enjoyment,^30^ administered by telehealth; and the Assessment of Life Habits,^28^ administered in‐person.

The Canadian Occupational Performance Measure,^22^, ^23^, ^25^, ^31^ Pediatric Evaluation of Disability Inventory,^25^ and Goal Attainment Scaling^25^ were used to assess activity and participation domains. The Canadian Occupational Performance Measure was administered both in‐person^22^, ^23^, ^25^ and by telehealth,^22^, ^31^ whereas the Pediatric Evaluation of Disability Inventory^25^ and Goal Attainment Scaling^25^ were used exclusively in‐person. The Pediatric Balance Scale^27^ assessed both the body structure and function domain and the activity domain and was administered in‐person. The Melbourne Assessment of Unilateral Upper Limb Function Scale^8^, ^21^, ^23^ and Quality of Upper Extremity Skills Test^24^ assessed the body function and activity domains. The Melbourne Assessment was administered in‐person in two studies,^21^, ^23^ whereas in another study the mode of administration was not reported^8^ and the Quality of Upper Extremity Skills Test was administered in‐person.^24^ The Participation and Environment Measure ‐ Children and Youth,^21^ administered in‐person, assessed both the participation domain and environmental factors.

**FIGURE 1:**
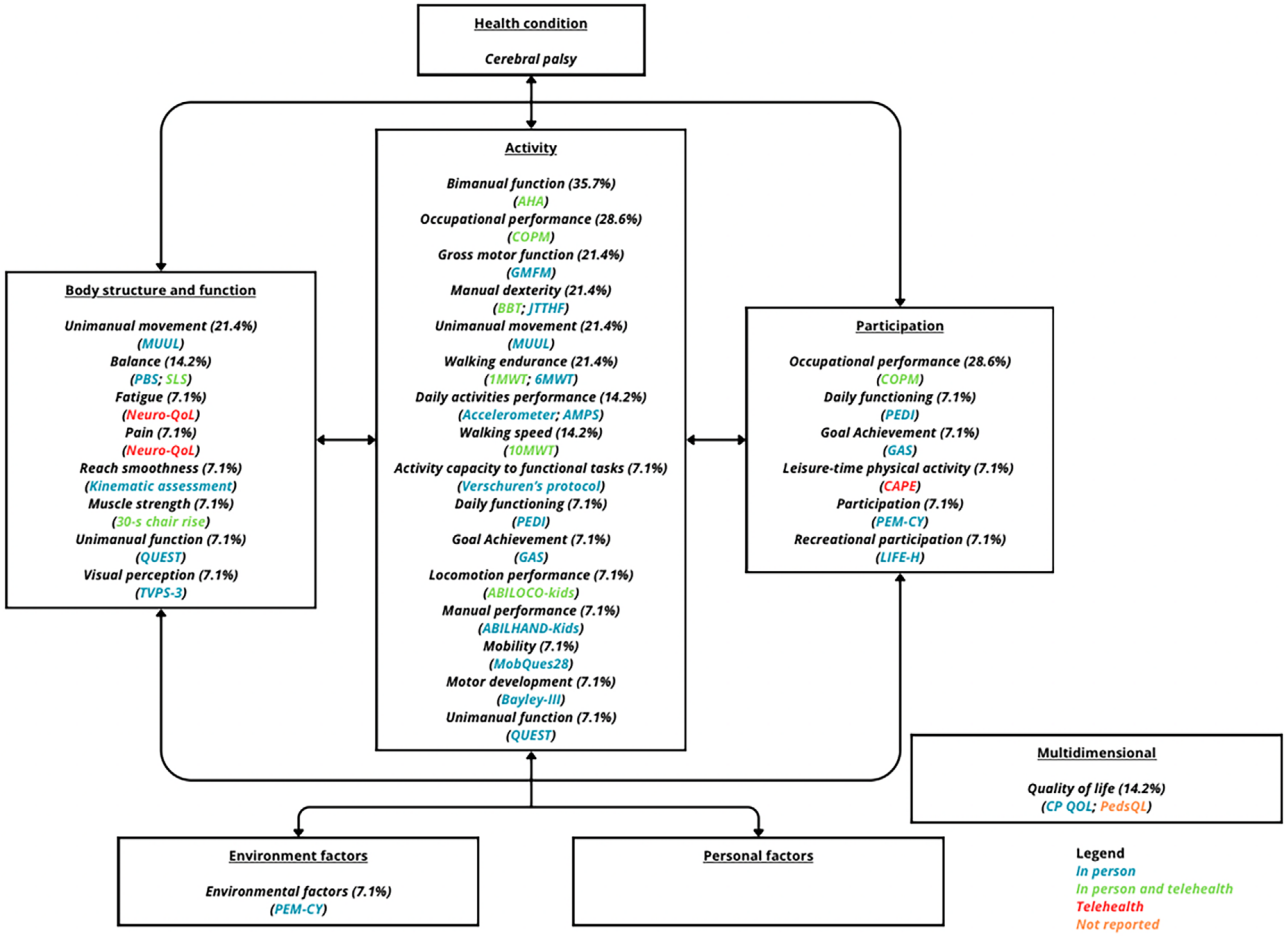
Summary of the outcomes, standardized tools, and mode of use. Abbreviations: 10MWT, 10‐metre walk test; 1MWT, 1‐minute walk test; 6MWT, 6‐minute walk test; AHA, Assisting Hand Assessment; AMPS, Assessment of Motor and Process Skills; Bayley‐III, Bayley Scales of Infant and Toddler Development, Third Edition; BBT, Box and Blocks Test; CAPE, Children's Assessment of Participation and Enjoyment; COPM, Canadian Occupational Performance Measure; CP QOL, Cerebral Palsy Quality of Life Questionnaire; GAS, Goal Attainment Scaling; GMFM, Gross Motor Function Measure; JTTHF, Jebsen–Taylor Test of Hand Function; MobQues28, 28‐item Mobility Questionnaire; LIFE‐H, Assessment of Life Habits; MUUL, Melbourne Assessment of Unilateral Upper Limb Function Scale; PBS, Pediatric Balance Scale; PEDI, Pediatric Evaluation of Disability Inventory; PedsQL, Pediatric Quality of Life Inventory; PEM‐CY, Participation and Environment Measure ‐ Children and Youth; QUEST, Quebec User Evaluation of Satisfaction with Assistive Technology; SLS, single leg stance; TVPS‐3, Test of Visual‐Perceptual Skills.

Two tools used to assess quality of life were classified as multidimensional: the Cerebral Palsy Quality of Life Questionnaire,^21^ administered in‐person; and the Pediatric Quality of Life Inventory,^7^ for which the mode of administration was not reported. A summary of the outcomes, standardized tools, and their modes of administration is presented in Table 2 and Figure **1**.

## DISCUSSION

This study identified outcomes and standardized tools used to measure changes in telehealth‐delivered physical therapy interventions for children and young people with CP up to 20 years of age. Our scoping review identified 27 outcomes, which were measured using 30 different standardized tools. The outcomes assessed by the standardized tools were predominantly focused on the activity domain. Although all interventions were delivered by telehealth, most studies still conducted assessments in‐person.

Activity‐domain outcomes are recognized as the primary priorities of parents of children and young people with CP,^32^ which probably explains their predominance among the outcomes identified in this review. The principal outcomes evaluated in the activity domain included bimanual function, manual dexterity, gross motor function, and walking endurance. These outcomes are frequently investigated in studies examining the effectiveness of interventions for children with CP^33^ and are related to the goals identified by families seeking physical therapy services.^33^, ^34^ Among these, bimanual function emerged as the most commonly assessed outcome, typically measured using the Assisting Hand Assessment.^8^, ^21^, ^22^, ^23^, ^24^ This is a widely used tool for children with unilateral CP and is considered a valid and reliable measure when administered in‐person.^21^, ^23^, ^24^ Manual dexterity was assessed by the Jebsen–Taylor Test of Hand Function in‐person^23^ and by the Box and Blocks Test in both modes.^21^, ^22^ In the study by Ferre et al.,^22^ an experienced evaluator conducted the in‐person assessment 1 week before the intervention, while caregivers were trained over two 1‐hour sessions to administer the assessment tools. The findings revealed no significant differences between the two modes of administration, with assessment scores for both the Assisting Hand Assessment and Box and Blocks Test demonstrating a high degree of correlation. These findings suggest that the Assisting Hand Assessment and Box and Blocks Test may be feasible for telehealth assessment, although additional studies are needed to confirm their reliability and validity for this mode of assessment.

Gross motor function was measured by the GMFM‐66 or GMFM‐88, a valid and reliable tool,^35^ and all studies included in this review used the GMFM in‐person,^25^, ^26^, ^27^ as recommended by the tool's manual. Walking endurance was assessed by the 6MWT administered in‐person^27^, ^28^ and by the 10MWT in‐person^9^, ^27^ and by telehealth.^9^ In the study conducted by Surana et al.,^9^ the 1MWT, 10 MW, ABILOCO‐Kids, 30‐second chair rise test, and single leg stance were administered both in‐person and by telehealth before the intervention. No statistically significant differences were observed between the two modes of administration. The 1MWT and single leg stance showed moderate reliability, whereas the 10MWT, ABILOCO‐Kids, and 30‐second chair rise test demonstrated good reliability.^9^ Thus, the 1MWT, 10MWT, and ABILOCO‐Kids may be considered promising, reliable tools for assessing walking endurance, walking speed, and locomotion performance, respectively, in the activity domain by telehealth. Similarly, the 30‐second chair rise test and single leg stance may facilitate a reliable assessment of indirect muscle strength and balance, respectively, in the body structure and function domain through telehealth. Further studies are required to evaluate the validity of these measures when administered by telehealth.

Only two studies^30^, ^31^ reported conducting all assessments exclusively by telehealth, using patient‐reported outcome measures. In the study by Lai et al.,^30^ adolescents aged 10 to 19 years were instructed to complete the Children's Assessment of Participation and Enjoyment, to assess leisure‐time physical activity, and Neurology Quality of Life Measurement System, to assess pain and fatigue, by telehealth, with caregiver assistance as needed. In the study by Oliveira et al.,^31^ the Canadian Occupational Performance Measure was administered by telehealth to identify goals in the activity and participation domains, which was scored by families under professional guidance. Given its interview‐based format, the Canadian Occupational Performance Measure is well‐suited for implementation through telehealth. Findings from a systematic review suggest that assessments using questionnaires exhibit higher validity and reliability for telehealth applications than objective outcome measures based on clinical scoring scales.^11^ Assessments that require technical skills, specialized training, or specific equipment are particularly challenging to implement by telehealth. Consequently, certain standardized instruments must be administered in‐person, as they rely on physical interaction, specialized equipment, or hands‐on techniques.

In the studies included in this review, standardized tools such as the Assessment of Motor and Process Skills, Melbourne Assessment of Unilateral Upper Limb Function Scale, Jebsen–Taylor Test of Hand Function, Test of Visual Perceptual Skills, Verschuren's protocol, 6MWT, Bayley Scales of Infant and Toddler Development, Third Edition, Quality of Upper Extremity Skills Test, and Pediatric Balance Scale were administered exclusively in‐person. Assessments conducted in‐person help to ensure maximum standardization for follow‐up evaluations and mitigate potential biases by maintaining consistency in factors such as location, timing, and attire.^27^ Additionally, kinematic movement analysis, primarily used in research owing to the high cost and fixed location of the equipment (e.g. Vicon Motion Systems), necessitates in‐person evaluations and has limited applicability in clinical practice. In contrast, questionnaire‐based measures, such as the Assessment of Life Habits, 28‐item Mobility Questionnaire, Participation and Environment Measure ‐ Children and Youth, Cerebral Palsy Quality of Life Questionnaire, ABILHAND‐Kids, Pediatric Quality of Life Inventory, and Pediatric Evaluation of Disability Inventory, were also administered in‐person in the studies included in this review. However, it is important to note that these tools can be administered through interviews with the children's families or directly with the children and adolescents, making telehealth administration a viable option for future studies.

Conducting assessments by telehealth presents several challenges as in‐person interaction is essential for performing direct physical examinations.^36^ The absence of human elements such as touch, empathy, and the complex dynamics of human interaction, coupled with ineffective communication, have been identified as significant barriers.^37^ Furthermore, there is limited information on the measurement properties of standardized tools used in a telehealth context.^9^, ^37^, ^38^ Some modifications or new versions of the instruments may be considered in the future for assessments by telehealth. For example, in the study by Lai et al.,^38^ a shorter, modified version of the 6MWT was developed for telehealth application to address the space constraints often encountered in participants' homes: the home‐modified 6‐minute walk test. A modified version of the GMFM‐88, known as the Gross Motor Family Report, has also recently been developed and can be administered by parents by telehealth.^39^, ^40^ This tool was designed to assess the usual performance in real‐world environments, in contrast to previous versions of the GMFM, which are a standardized motor tools that evaluates the skills demonstrated in clinical environments (capacity).^40^ However, it has not yet been used in any telehealth intervention studies.

It is worth pointing out that some standardized tools were developed before the introduction of the ICF framework or were not designed to be confined to a single domain, which may present challenges in classification within the ICF model. In addition to the Canadian Occupational Performance Measure, instruments such as the Pediatric Evaluation of Disability Inventory, Goal Attainment Scaling, Melbourne Assessment of Unilateral Upper Limb Function Scale, Quality of Upper Extremity Skills Test, and Participation and Environment Measure ‐ Children and Youth can assess multiple domains within the ICF framework. Quality of life is a multidimensional concept encompassing the subjective perceptions of physical, emotional, mental, and social functioning; therefore it includes various domains of the ICF.^41^ In line with the definition, quality of life was assessed in studies using multidimensional tools: the Cerebral Palsy Quality of Life Questionnaire^21^ and Pediatric Quality of Life Inventory.^7^

Notably, the delivery of interventions by telehealth is not a recent development, because half (50%) of the studies included in this review were conducted before the COVID‐19 pandemic. The exponential growth of telehealth following the pandemic^12^ highlights the need to better understand how to appropriately assess individuals participating in these interventions. In a study by Hall et al.,^2^ 76% of physiotherapists recommended a hybrid model for delivering telehealth interventions in paediatric physiotherapy. A hybrid service delivery model, which incorporates in‐person assessments, can facilitate comprehensive evaluations across all domains of functionality. Therefore it is essential to identify standardized tools that are both valid and reliable for telehealth use, enabling comprehensive assessments for individuals who are unable to attend in‐person evaluations.

This scoping review encompassed a variety of physical therapy interventions, including motor‐learning‐based treatment,^4^ movement‐to‐music,^30^ lower‐extremity functional training,^9^ action observation therapy,^8^, ^21^, ^27^ multicomponent upper extremity intervention,^29^ goal‐oriented activities,^25^ intensive bimanual training,^22^ home programme,^31^ and Web‐based multimodal therapy.^23^, ^28^ This diversity reinforces the applicability of telehealth across different intervention types. Notably, several common components emerge across these interventions, such as goal‐directed practice for real‐life tasks, child‐initiated active movements, high‐intensity active participation, and family involvement in intervention planning.^33^ These components align with international clinical practice guidelines, which recommend active practice of the child's goals in home or community settings rather than focusing solely on underlying impairments.^42^ Delivering interventions by telehealth that incorporate family‐centred principles increases opportunities to practise tasks in natural environments,^31^ thereby enhancing the likelihood of long‐term skill transfer to daily life.^43^ Despite these advantages, participation outcomes remain under‐evaluated, probably because few interventions specifically target this domain.^33^, ^34^ Interventions addressing contextual factors are also limited, emphasizing their importance and the need for greater consideration in both research and clinical practice. Interventions such as environmental enrichment are designed to facilitate environmental adaptations or modifications, thereby increasing opportunities for practising motor skills.^33^ Consequently, modifiable environmental factors should be systematically assessed and monitored throughout the intervention process. In this review, only one study addressed environmental factors using the Participation and Environment Measure ‐ Children and Youth.

It is important to note that, although most studies included in the review were randomized controlled trials, studies with lower methodological rigor, such as non‐randomized designs and case series with small sample size, were also included, which may have introduced bias. Furthermore, some studies used standardized tools by telehealth without previous validation of their measurement properties for this mode of administration. In addition, aligning with previous research,^34^ these studies reveal that most interventions focus on children with CP classified in lower GMFCS levels. Notably, our review is the first study to provide data on the GMFCS classification of children and young people with CP included in physical therapy interventions by telehealth studies.^44^, ^45^ This highlights the need for further research to assess the effectiveness of physical therapy interventions by telehealth for children with CP classified in GMFCS levels III, IV, and V.

In addition to the expert knowledge of clinicians working with this population, future studies could help us identify which outcomes are most relevant for families as well as those that are important to children and young people with CP. The results found by this scoping review will contribute to the development of a set of core outcomes for telehealth‐delivered physical therapy interventions for children with CP. The adoption of a set of core outcomes facilitates the standardization of key outcomes that are significant to clinicians, patients, and their families, ensuring these outcomes are recommended for use in randomized clinical trials.^46^, ^47^, ^48^

## CONCLUSION

The findings from this scoping review indicate that the outcomes and standardized tools used to assess changes after telehealth‐delivered physical therapy interventions in children and young people with CP up to 20 years of age are aligned with ICF domains. Notably, these assessments are predominantly applied in the in‐person mode. Since telehealth offers the opportunity to observe an individual's performance in a natural environment, future studies may examine the use of standardized tools for this purpose, for the population of children and young people with CP. Further studies are needed to evaluate the feasibility and measurement properties of outcome and standardized tools used in telehealth settings.

## CONFLICT OF INTEREST STATEMENT

The authors have stated that they had no interests that might be perceived as posing a conflict or bias.

## Supporting information


**Figure S1:** Flowchart adapted from the PRISMA‐ScR for study selection.

**Table S1:** Search strategy conducted in November 2024.

## Data Availability

Data available on request from the authors.
