# Supplementary figures and images for: Outcomes and standardized tools in telehealth physical therapy for children with cerebral palsy: A scoping review using the ICF framework
Source: Dev Med Child Neurol. 2025 Oct 17;68(4):477–88. doi: 10.1111/dmcn.70006 (PMC12982665; doi:10.1111/dmcn.70006)

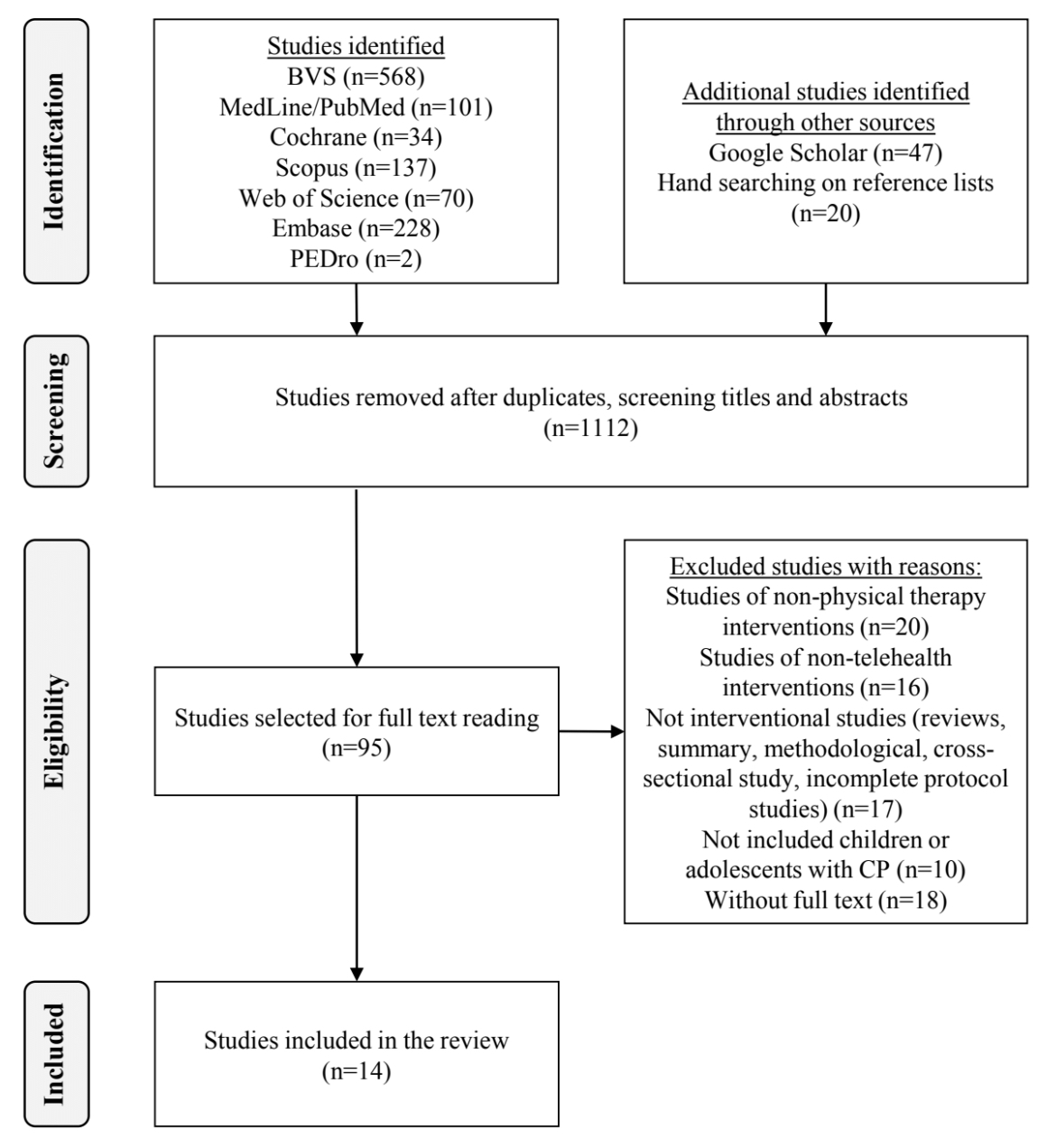

Supplement: Supplementary file 1 — Figure S1: Flowchart adapted from the PRISMA‐ScR for study selection. [file DMCN-68-477-s001.tiff]
