# Supplementary material for: Outcomes and standardized tools in telehealth physical therapy for children with cerebral palsy: A scoping review using the ICF framework
Source: Dev Med Child Neurol. 2025 Oct 17;68(4):477–88. doi: 10.1111/dmcn.70006 (PMC12982665; doi:10.1111/dmcn.70006)
Supplement: Supplementary file 2 — Table S1: Search strategy conducted in November 2024. [file DMCN-68-477-s002.docx]

Supplementary Table 1. Search strategy conducted in November 2024.

| **Database** | **Research Strategy** |
| --- | --- |
| BVS | ("Paralisia Cerebral" OR "Cerebral Palsy" OR "Parálisis Cerebral" OR "Paralysie cérébrale") AND ("Especialidade de Fisioterapia" OR "Physical Therapy Specialty" OR "Especialidad de Fisioterapia" OR "Kinésithérapie (spécialité)" OR fisioterapeutas OR "Physical Therapists" OR fisioterapeutas OR kinésithérapeutes OR reabilitação OR rehabilitation OR rehabilitación OR réadaptation OR "Terapia por Exercício" OR "Exercise Therapy" OR "Terapia por Ejercicio" OR "Traitement par les exercices physiques" OR "Exercício Terapêutico" OR "Exercício de Reabilitação" OR "Reabilitação Neurológica" OR "Neurological Rehabilitation" OR "Rehabilitación Neurológica" OR "Rééducation neurologique" OR "Rehabilitation Exercise") AND (telemedicina OR telemedicine OR télémédecine OR telessaúde OR e-saúde OR esaúde OR msaúde OR telehealth OR ehealth OR mhealth) AND ( db:("LILACS" OR "IBECS" OR "WPRIM" OR "BDENF" OR "INDEXPSI" OR "tese" OR "BBO" OR "BINACIS" OR "BRISA" OR "CUMED" OR "AIM" OR "ARGMSAL" OR "BIGG" OR "CidSaude" OR "HomeoIndex" OR "LIPECS" OR "LIS" OR "MedCarib")) |
| Medline/ Pubmed | ("Cerebral Palsy") AND ("Physical Therapy Specialty" OR "Physical Therapists" OR Rehabilitation OR "Exercise Therapy" OR "Neurological Rehabilitation" OR "Rehabilitation Exercise") AND (Telemedicine OR Telehealth OR eHealth OR mHealth) |
| Cochrane | ("Cerebral Palsy") AND ("Physical Therapy Specialty" OR "Physical Therapists" OR Rehabilitation OR "Exercise Therapy" OR "Neurological Rehabilitation" OR "Rehabilitation Exercise") AND (Telemedicine OR Telehealth OR eHealth OR mHealth) |
| Scopus | ("Cerebral Palsy") AND ("Physical Therapy Specialty" OR "Physical Therapists" OR Rehabilitation OR "Exercise Therapy" OR "Neurological Rehabilitation" OR "Rehabilitation Exercise") AND (Telemedicine OR Telehealth OR eHealth OR mHealth) |
| Web of Science | ("Cerebral Palsy") AND ("Physical Therapy Specialty" OR "Physical Therapists" OR Rehabilitation OR "Exercise Therapy" OR "Neurological Rehabilitation" OR "Rehabilitation Exercise") AND (Telemedicine OR Telehealth OR eHealth OR mHealth) |
| Embase | ('cerebral palsy') AND (physiotherapy OR physiotherapist OR rehabilitation OR kinesiotherapy OR neurorehabilitation) AND (telemedicine OR telehealth) |
| Pedro | Cerebral Palsy AND Telehealth |
| Google Scholar | Cerebral Palsy physiotherapy Telehealth |
